# Changes in Expression of the *CLOCK* Gene in Obstructive Sleep Apnea Syndrome Patients Are Not Reverted by Continuous Positive Airway Pressure Treatment

**DOI:** 10.3389/fmed.2017.00187

**Published:** 2017-11-06

**Authors:** Susana Moreira, Raquel Rodrigues, André B. Barros, Nadja Pejanovic, Ana Neves-Costa, Dora Pedroso, Cláudia Pereira, Dina Fernandes, João Valença Rodrigues, Cristina Barbara, Luís Ferreira Moita

**Affiliations:** ^1^Departamento de Pneumologia, Centro Hospitalar Lisboa Norte, EPE, Lisboa, Portugal; ^2^Instituto Gulbenkian de Ciência, Oeiras, Portugal; ^3^Faculdade de Medicina, Instituto de Medicina Molecular, Universidade de Lisboa, Lisboa, Portugal

**Keywords:** obstructive sleep apnea syndrome, clock genes, continuous positive airway pressure, slow wave sleep, Metabolic Syndrome, Circadian Rhythms

## Abstract

**Purpose:**

Metabolic syndrome and cardiovascular disease are strongly associated with obstructive sleep apnea syndrome (OSAS), which causes substantial changes to normal circadian physiological functions, including metabolic pathways. Because core clock genes are known to be modulated by sleep/vigilance cycles, we asked whether the expression level of mRNA coding for clock genes is altered in non-treated OSAS patients and if it can be corrected by standard continuous positive airway pressure (CPAP) treatment.

**Methods:**

Peripheral blood was collected from male patients diagnosed with severe OSAS (apnea-hypopnea index ≥ 30/h) before and after treatment initiation. qPCR was used to measure mRNA levels of genes associated with the central circadian pacemaker including *CLOCK, BMAL1, Cry1, Cry2*, and three *Period* genes (*Per 1, 2, 3*) in peripheral blood mononuclear cells (PBMCs).

**Results:**

We found statistically significant differences for *CLOCK* (*p*-value = 0.022) expression in PBMCs of OSAS patients which were not reverted by treatment with CPAP. We have also found a substantial decrease in the slow wave sleep (SWS) content in OSAS patients (*p*-value < 0.001) that, contrary to REM sleep, was not corrected by CPAP (*p*-value = 0.875).

**Conclusion:**

CPAP treatment does not correct substantial changes in expression of core clock genes in OSAS patients. Because CPAP treatment is also unable to normalize the SWS in these patients, it is likely that additional therapeutic interventions that increase SWS content and complement the benefits of CPAP are required to more effectively reduce the known increased cardiovascular risk associated with OSAS patients.

## Introduction

The obstructive sleep apnea syndrome (OSAS) is a frequent sleep disorder that constitutes an independent risk factor for the development of metabolic syndrome and cardiovascular disease (1). Most of the gene components of critical metabolic pathways and their regulators exhibit circadian variation at the mRNA and protein levels (2). They are regulated by core clock genes, which in turn are modulated by sleep/vigilance cycles (3). Importantly, single genetic disruption of the core clock genes *CLOCK* (4) and *BMAL1* (5, 6) cause full-blown metabolic syndrome phenotypes in mice. Milder metabolic syndrome phenotypes have also been observed in double or triple, but not single, inactivation of additional core clock genes, including *Per1, 2, 3*, and *Cry1 and 2* (7). In this work, we investigated whether the expression level of mRNA coding for clock genes is altered in non-treated OSAS patients and if it can be corrected by continuous positive airway pressure (CPAP) treatment.

## Materials and Methods

### Study Population

Using polysomnography (Alice 5, Respironics, Inc., Murrysville, PA, USA) scored according to the 2007 AASM manual for the scoring of sleep and associated events and stringent clinical criteria (patients with addiction habits, cancer, hematological disorders, active infection, or shift work were excluded), we have identified a group of 13 male patients with severe OSAS [average apnea-hypopnea index (AHI) of 66.7/h, all ≥ 30/h] and 7 matched controls for sex (average AHI of 2, all < 5/h). Patients and controls, without the exclusion criteria, were recruited at the sleep consultation of Santa Maria Hospital (Lisbon, Portugal), from those with a high degree of suspicion to have Sleep Apnea, based on their clinical history. Patients and controls were distinguished on the basis of the PSG results. Sleepiness was evaluated using the Stanford Sleepiness Scale and the Epworth Sleepiness Scale (ESS). See Table 1 for sleep study results in the healthy and OSAS group. Cortisol levels were within normal limits for all subjects (data not shown). For a subset of patients, PSG was repeated after treatment with CPAP to assess its impact on REM and slow wave sleep (SWS) content. This study was approved by the Ethics Committee Review Board of Centro Hospitalar Lisboa Norte, EPE (Lisboa, Portugal), and written consent obtained for every patient and control sample included in this study.

**Table 1:** Polysomnography characterization of healthy controls and obstructive sleep apnea syndrome patients, before continuous positive airway pressure treatment.

	Control group				Patient group			

*N*	7				13			
	1st Quartile	Median	Mean	3rd Quartile	1st Quartile	Median	Mean	3rd Quartile
Age (years)	35.50	40.00	44.57	54.50	46.00	51.00	49.46	54.00
Oxygen desaturation index	0.10	0.30	1.16	1.60	40.60	59.70	57.92	71.40
Time spent with SpO_2_ < 90%, (min)	0.00	0.00	0.21	0.00	17.80	84.40	103.10	146.00
Lowest SpO_2_ (%)	91.25	92.50	91.50	93.00	68.00	75.00	72.69	78.00
Mean SpO_2_ (%)	96.00	96.00	96.00	96.00	91.00	91.00	91.23	93.00
ESS	0.00	2.00	2.60	3.00	9.00	12.00	11.45	15.00
Sleep efficiency (%)	76.70	90.00	84.69	94.30	72.72	82.60	80.34	88.92
Total sleep time (min)	366.40	377.20	368.70	389.20	295.80	353.20	350.00	399.50
REM (min)	40.12	60.25	58.48	76.62	3.65	8.45	9.40	15.62
Slow wave sleep (min)	53.50	58.25	58.75	75.38	0.00	1.55	3.86	4.63
Arousal index (events/h)	2.70	7.20	5.32	7.20	37.25	55.05	57.52	72.35
Apnea-hypopnea index (events/h)	0.85	1.50	2.00	3.05	41.50	67.10	61.88	78.40

### Measurement of Gene Expression

We have used quantitative RT-PCR to systematically quantify the expression of the core clock genes in peripheral blood cells of severe OSAS patients in comparison with that of matched non-affected control volunteers, before and after 1 month of treatment with CPAP. Several studies have demonstrated that clock gene expression profiles in human peripheral blood mononuclear cells (PBMCs) can be used as surrogate measures of central circadian rhythm expression changes in patients and healthy subjects (8–11). Therefore, peripheral blood was collected from the study subjects before and after 1 month of treatment initiation. Collections were always performed between 8 and 10 a.m. Blood was then used to perform routine biochemical analyses (including cortisol levels) and to isolate PBMCs. The following hematologic and biochemical parameters were investigated in all participants: full peripheral blood cell count; hepatic enzymes (aspartate transaminase, alanine transaminase, and gamma-glutamyltransferase); renal parameters (creatinine, blood urea nitrogen); standard metabolic parameters (uric acid, glucose, total cholesterol, low density lipoprotein, high density lipoprotein, triglycerides, and C-reactive protein). RNA was isolated and quantitative RT-PCR used to measure mRNA levels of genes associated with the central circadian pacemaker including *CLOCK, BMAL1*, two *Cry* (*Cry 1, 2*), and three *Period* genes (*Per 1, 2, 3*). The selected patients were then evaluated 1 month after therapy initiation with CPAP and the mRNA level of the same genes tested. The sequence of the oligos used for RT-PCR can be found Table S2 in the Supplementary Material.

### Statistical Analysis

A non-parametric approach was chosen for the data analysis. We used median and quartiles for the descriptive statistical analysis. To test hypotheses related to differences between the control group and the patient group, we used the Wilcoxon signed-rank test. To investigate relationships between gene expression and other parameters, we implemented a correlation analysis based on Spearman’s rank correlation coefficient.

## Results

Using Wilcoxon signed-rank test, we found statistically significant differences between patients and control volunteers in the ESS (*W* = 51.5, *p*-value = 0.007) and in polysomnography parameters (Table 1) including in AHI (*W* = 91, *p*-value < 0.0001), oxygen desaturation index (*W* = 91, *p*-value < 0.001), SpO_2_ time < 90% in minutes (*W* = 90, *p*-value < 0.001), minimal SpO_2_ (*W* = 1, *p*-value < 0.0001), and average SpO_2_ (*W* = 1.5, *p*-value < 0.001). Statistically significant differences were also found for SWS in minutes (*W* = 0, *p*-value < 0.001), REM sleep in minutes (*W* = 0, *p*-value = 0.0001), and arousal index (*W* = 60, *p*-value < 0.002), but not for sleep efficiency (*W* = 29.5, *p*-value = 0.310) or total sleep time (*W* = 34, *p*-value = 0.892).

On the comparison between control volunteers and patients before treatment initiation, we found statistically significant differences for *CLOCK* (*W* = 15, *p*-value = 0.022) expression in PBMCs of patients, but not for *Cry 1* and *2* or the three *Period* genes tested (Table 2). The differences of *BMAL1* expression between control volunteers and patients nearly reached statistical significance (*W* = 20, *p*-value = 0.068), an outcome that is likely, should the population size be larger.

**Table 2:** Characterization of the gene expression between the control group (C) and the patient group at t1 (P_t1_), as well as the results of hypothesis tests for comparison of gene expression between these groups.

		Min	1st Quartile	Median	3rd Quartile	Max	Mean	SD	Hypothesis test
BMAL1	P_t1_	0.54	0.72	0.81	1.27	5.01	1.26	1.22	*W* = 20, *p*-value = 0.068
	C	0.91	1.09	1.38	1.95	2.27	1.52	0.54	
CLOCK	P_t1_	0.16	0.42	0.99	1.32	2.53	1.03	0.69	*W* = 15, *p*-value = 0.022
	C	1.18	1.29	2.20	2.31	3.72	2.04	0.90	
CRY1	P_t1_	0.96	1.18	1.52	2.12	2.85	1.68	0.65	*W* = 22, *p*-value = 0.230
	C	1.26	1.58	2.13	2.57	3.31	2.14	0.73	
CRY2	P_t1_	0.19	1.30	1.53	1.95	3.08	1.58	0.79	*W* = 41, *p*-value = 0.601
	C	0.82	1.01	1.33	1.81	1.97	1.39	0.47	
PER1	P_t1_	0.05	0.32	0.40	0.59	0.98	0.48	0.29	*W* = 27, *p*-value = 0.591
	C	0.26	0.37	0.61	0.80	0.85	0.58	0.25	
PER2	P_t1_	0.83	1.58	2.61	2.88	3.51	2.35	0.88	*W* = 35, *p*-value = 0.438
	C	2.06	2.26	2.52	3.98	6.20	3.32	1.63	
PER3	P_t1_	1.20	2.45	2.83	4.42	11.46	4.52	3.70	*W* = 53, *p*-value = 0.088
	C	1.77	2.22	2.27	2.59	3.74	2.48	0.62	

*Only the CLOCK gene showed statistically significant changes*.

Patient treatment with CPAP did not change abnormal expression of clock genes (Table 3). It also did not improve the reduced levels of SWS observed in non-treated OSAS patients (*p*-value = 0.875, Table 4), contrary to REM sleep. The persistence of the abnormal expression pattern of the *CLOCK* gene cannot be attributed to the lack or sub-optimal adherence levels to CPAP treatment as demonstrated by the metrics of CPAP adherence of our patients (Table 5; Table S1 in Supplementary Material). Our data show that adherence to CPAP treatment is well above the minimum required level recommended by the most recent proposal of the American Thoracic Society (12) that points to the need of CPAP use in more than 70% of nights and for more than 4 h per night.

**Table 3:** Characterization of the gene expression within the patient group between the values recorded at t1 (P_t1_) and the ones recorded at t2 (P_t2_), as well as the results of hypothesis tests for comparison of gene expression between these time-points.

		Min	1st Quartile	Median	3rd Quartile	Max	Mean	SD	Hypothesis test
BMAL1	P_t1_	0.54	0.72	0.81	1.27	5.01	1.26	1.22	*W* = 57, *p*-value = 0.872
	P_t2_	0.21	0.87	1.13	1.28	1.65	1.03	0.45	
CLOCK	P_t1_	0.16	0.42	0.99	1.32	2.53	1.03	0.69	*W* = 58, *p*-value = 0.923
	P_t2_	0.14	0.87	0.98	1.20	1.95	1.07	0.54	
CRY1	P_t1_	0.96	1.18	1.52	2.12	2.85	1.68	0.65	*W* = 32, *p*-value = 0.813
	P_t2_	0.85	1.20	1.55	2.38	3.86	1.92	1.06	
CRY2	P_t1_	0.19	1.30	1.53	1.95	3.08	1.58	0.79	*W* = 36, *p*-value = 0.756
	P_t2_	0.87	1.23	1.46	2.65	4.03	2.04	1.29	
PER1	P_t1_	0.05	0.32	0.40	0.59	0.98	0.48	0.29	*W* = 50, *p*-value = 1.000
	P_t2_	0.21	0.37	0.39	0.50	0.74	0.43	0.17	
PER2	P_t1_	0.83	1.58	2.61	2.88	3.51	2.35	0.88	*W* = 65, *p*-value = 0.733
	P_t2_	1.07	2.24	2.74	2.79	5.42	2.70	1.11	
PER3	P_t1_	1.20	2.45	2.83	4.42	11.46	4.52	3.70	*W* = 27, *p*-value = 0.475
	P_t2_	2.42	2.94	4.36	4.96	8.44	4.43	2.07	

*These comparisons did not reveal any significant differences*.

**Table 4:** Slow wave sleep (SWS) and REM content, shown in percentage (%), in controls and patients (at diagnosis and after continuous positive airway pressure treatment).

	Patients				Controls	
	No treatment		Treatment			

	REM	SWS	REM	SWS	REM	SWS
Min	0.00	0.00	9.60	1.40	9.50	3.80
1st Q	3.68	2.25	12.53	3.28	12.05	10.20
Median	6.70	4.45	15.30	4.35	15.90	15.60
Mean	7.85	5.48	16.85	4.95	15.53	14.86
3rd Q	10.88	7.68	19.62	6.03	17.95	20.00
Max	18.00	13.00	27.20	9.70	23.30	24.20

**Table 5:** Statistical Characterization of the continuous positive airway pressure adherence.

	Min	1st Quartile	Median	Mean	3rd Quartile	Max
Days of use (%)	76	86.8	96.8	93.1	100.0	100.0
Average number of hours of use per day (h)	1.1	3.35	5.42	4.97	6.60	7.37
IAH flow	0.5	1.15	1.95	3.07	3.55	10.40
IA flow	0.3	0.45	0.70	1.43	1.68	6.70

## Discussion

In our study, as expected, control volunteers and OSAS patients could be easily distinguished by the statistical analysis of the ESS results and polysomnography parameters. They could also be distinguished on the basis of expression levels of *CLOCK*, but not any of the other core clock genes, with the possible exception of *BMAL1*. Interestingly, these results are strikingly reminiscent of metabolic syndrome phenotypes found in the knockout of the corresponding genes in mice as described earlier. The levels of expression of the *CLOCK* gene were altered in OSAS patients before and after CPAP treatment, suggesting that either the standard therapeutic management of these patients is insufficient to correct the disruption of the circadian patterns of genes with expression conditioned by cycles of sleep/vigilance or changes in severe sleep apnea patients are structural, for example, as a consequence of irreversible cerebral vascular lesions.

Before patients were treated, we observed consistently and substantially reduced levels of REM and SWS content as compared to healthy controls. Perhaps significantly, while all the patients who initiated CPAP recovered to normal levels of REM sleep, SWS content remained invariably abnormal, at much lower levels than controls (Table 4). It was previously shown that even in young healthy adults, selective suppression of SWS causes pronounced decrease of insulin sensitivity, reduced glucose tolerance, and higher diabetes risk (13).

While it is clear that CPAP substantially improves cognitive and behavioral aspects of the disease (14), metabolic parameters and other OSAS-related risk factors are only partially modified (15), which might explain the incomplete reduction of cardiovascular risk in OSAS. These facts point to the need to complement CPAP therapy with other therapy interventions that are aimed at the sleep structure and circadian rhythm normalization and are likely to have a substantial impact on the natural history of the disease. Drugs that effectively improve the SWS content of patients might constitute prime candidates for this goal.

### Study Limitations

While our major conclusions are supported by robust statistically significant results, our study would benefit from increasing the size of the population of both patients and controls. It is likely that with more samples, statistically significant associations will be found for additional clock genes, especially in the case of *BMAL1*, where a trend is already clear with the current analysis.

## Informed Consent

Informed consent was obtained from all individual participants included in the study.

## Ethics Statement

All procedures performed in studies involving human participants were in accordance with the ethical standards of the institutional (Centro Hospitalar Lisboa Norte, EPE) and national research committee and with the 1964 Helsinki Declaration and its later amendments or comparable ethical standards.

## Author Contributions

SM and LM conceived and designed the study; SM, RR, NP, ANC, DP, CP, DF, JR, and CB collected the data. RR, AB, and LM analyzed the data. LM wrote the manuscript.

## Conflict of Interest Statement

All authors certify that they have no affiliations with or involvement in any organization or entity with any financial interest (such as honoraria; educational grants; participation in speakers’ bureaus; membership, employment, consultancies, stock ownership, or other equity interest; and expert testimony or patent-licensing arrangements), or non-financial interest (such as personal or professional relationships, affiliations, knowledge or beliefs) in the subject matter or materials discussed in this manuscript. The reviewer J H-R and handling editor declared their shared affiliation.
