# Supplementary material for: Changes in Expression of the CLOCK Gene in Obstructive Sleep Apnea Syndrome Patients Are Not Reverted by Continuous Positive Airway Pressure Treatment
Source: Front Med (Lausanne). 2017 Nov 6;4:187. doi: 10.3389/fmed.2017.00187 (PMC5681745; doi:10.3389/fmed.2017.00187)
Supplement: Supplementary file 1 [file data_sheet_1.docx]

**Supplementary Material**

**Table S1.** Raw results and Statistical Analysis on CPAP Treatment

| **Patients** | **Days of Use (%)** | **Average Number of Hours of Use *per* Day (h)** | **IAH Flow** | **IA Flow** |
| --- | --- | --- | --- | --- |
| PS1 | 100.00 | 6.98 | 3.40 | 2.90 |
| PS2 | 100.00 | 5.45 | 1.10 | 0.70 |
| PS3 | 100.00 | 5.77 | 7.80 | 6.70 |
| PS4 | 96.90 | 3.05 | 2.00 | 0.90 |
| PS5 | 96.70 | 2.60 | 1.30 | 0.40 |
| PS6 | 86.70 | 4.93 | 3.60 | 1.00 |
| PS7 | 80.00 | 1.10 | 10.40 | 0.70 |
| PS8 | 100.00 | 4.23 | 0.50 | 0.30 |
| PS9 | 87.00 | 5.38 | 0.90 | 0.60 |
| PS10 | 94.20 | 6.80 | 2.60 | 1.90 |
| PS11 | NA | NA | NA | NA |
| PS12 | 100.00 | 7.37 | 1.30 | 0.30 |
| PS13 | 76.00 | 6.00 | 1.90 | 0.70 |
| **Min** | 76.00 | 1.10 | 0.50 | 0.30 |
| **1st Quartile** | 86.78 | 3.35 | 1.15 | 0.45 |
| **Median** | 96.80 | 5.42 | 1.95 | 0.70 |
| **Mean** | 93.13 | 4.97 | 3.07 | 1.43 |
| **3rd Quartile** | 100.00 | 6.60 | 3.55 | 1.68 |
| **Max** | 100.00 | 7.37 | 10.40 | 6.70 |

**Table S2 – Oligos used for the qPCR determination**

| **CLOCK_F** | TGCGAGGAACAATAGACCCAA |
| --- | --- |
| **CLOCK_R** | ATGGCCTATGTGTGCGTTGTA |
| **PER1_F** | GCTCCTCCTCAGGCAACG |
| **PER1_R** | TCAGGAGGCTGTAGGCAATG |
| **PER2_F** | GGGCAGTGACTGTGACGAC |
| **PER2_R** | CGCTACTGCAGCCACTTGTA |
| **PER3_F** | GCAGAGGAAATTGGCGGACA |
| **PER3_R** | GGTTTATTGCGTCTCTCCGAG |
| **BMAL1** | CCAACATGCAACGCAATG |
| **BMAL2** | GGATTGGTGGCACCTCTTAAT |
| **CRY1_F** | CTCCTCCAATGTGGGCATCAA |
| **CRY1_R** | CCACGAATCACAAACAGACGG |
| **CRY2_F** | TCCCAAGGCTGTTCAAGGAAT |
| **CRY2_R** | TGCATCCCGTTCTTTCCCAAA |
